# Case report: Lichenoid eruption under immunotherapy with MK-4830 and pembrolizumab in a breast cancer patient

**DOI:** 10.3389/fphar.2024.1445685

**Published:** 2024-08-13

**Authors:** Zofia Kachlik, Izabela Błażewicz, Aleksandra Ciarka, Roman J. Nowicki

**Affiliations:** ^1^ Department of Dermatology, Venereology and Allergology, Faculty of Medicine, Medical University of Gdansk, Gdansk, Poland; ^2^ Department of Pathomorphology, Medical University of Gdansk, Gdansk, Poland

**Keywords:** lichenoid eruption, lichen planus, immune-related adverse events, immune checkpoint inhibitors, PD-1 inhibitor, MK-4830, pembrolizumab, breast cancer

## Abstract

**Background:**

Immune checkpoint inhibitors (ICIs) have revolutionized cancer treatment, yet they can induce immune-related adverse events (irAEs), including cutaneous toxicities such as lichenoid eruptions. Pembrolizumab, a PD-1 inhibitor, is known for its association with lichen-planus-like reactions, while the side effect profile of combining immunotherapy with MK-4830, a novel fully human IgG4 monoclonal antibody that targets ILT-4, remains limited.

**Case report:**

We present a case of a 47-year-old female with metastatic breast cancer who developed a grade 2 Common Terminology Criteria for Adverse Events (CTCAE) lichenoid reaction after 9 months of MK-4830 and pembrolizumab use. Confluent, erythematous papules with Wickham’s striae appeared predominantly on the extremities. Initial therapy with high-potency topical corticosteroids proved insufficient, however prednisone 40 mg daily resulted in satisfactory remission of lichen-planus-like reaction, permitting continued immunotherapy without dosage adjustment.

**Conclusion:**

This case highlights the novel occurrence of lichenoid eruption induced by MK-4830 and pembrolizumab in breast cancer treatment. The patient was successfully treated with oral prednisone, which controlled the skin symptoms without interrupting ICI therapy. We emphasize that early diagnosis and treatment of low-grade lichenoid eruption can prevent the cessation of ICIs, thereby combining the benefits of managing irAEs and avoiding cancer progression, leading to a better long-term prognosis.

## Introduction

Over the last decade, immune checkpoint inhibitors (ICIs), including monoclonal antibodies against programmed death 1 (PD-1) or its ligand, programmed death ligand 1 (PD-L1), have markedly transformed cancer treatment; however, many patients do not respond to these therapies, or they eventually develop resistance to them. Targeting distinct mechanisms via combining immunotherapies may enhance outcomes and overcome resistance. In this context, efforts have been made to enhance stimulation of the antitumor T-cell response with anti-PD-1 therapy by introducing an agent capable of reprogramming myeloid-derived suppressor cells (MDSCs), and therefore contribute to immunosuppression in the tumor microenvironment (Siu et al., 2022). MK-4830, a novel first-in-class human IgG4 monoclonal antibody targeting the immunoglobulin-like transcript 4 (ILT4) receptor, is being introduced as such an agent, as it induces a proinflammatory response by inhibiting ILT4 in MDSCs, thereby enhancing the T-cell response with anti-PD-1 therapy (Siu et al., 2022).

While ICIs offer tremendous potential for direct clinical benefits, they can also non-specifically activate the immune system, leading to a subtype of side effects known as immune-related adverse events (irAEs). Cutaneous toxicities are among the most common irAEs, occurring in 30%–40% of patients treated with PD-1 inhibitors, and include a variety of conditions such as nonspecific maculopapular rash, pruritus, psoriasiform, eczematous, and lichenoid dermatoses (Geisler et al., 2020). The prevalence of anti-PD-1 induced lichenoid eruptions is estimated to range from 0.5% to 6%; however, it is likely that lichen planus-like reactions following PD-1 inhibitors are underestimated due to their sporadic reporting in clinical literature (Curry et al., 2017; Phillips et al., 2019). Although pembrolizumab, a PD-1 inhibitor, has been frequently linked to lichen-planus-like reactions, there is limited data on the side effect profile of combination immunotherapy with MK-4830. We report a case of drug-induced lichenoid eruption secondary to immunotherapy with MK-4830 in combination with pembrolizumab in a patient with metastatic breast cancer. To our knowledge, this is the first case describing a lichen-planus-like reaction in a patient receiving MK-4830 in combination with pembrolizumab.

## Case report

**FIGURE 1:**
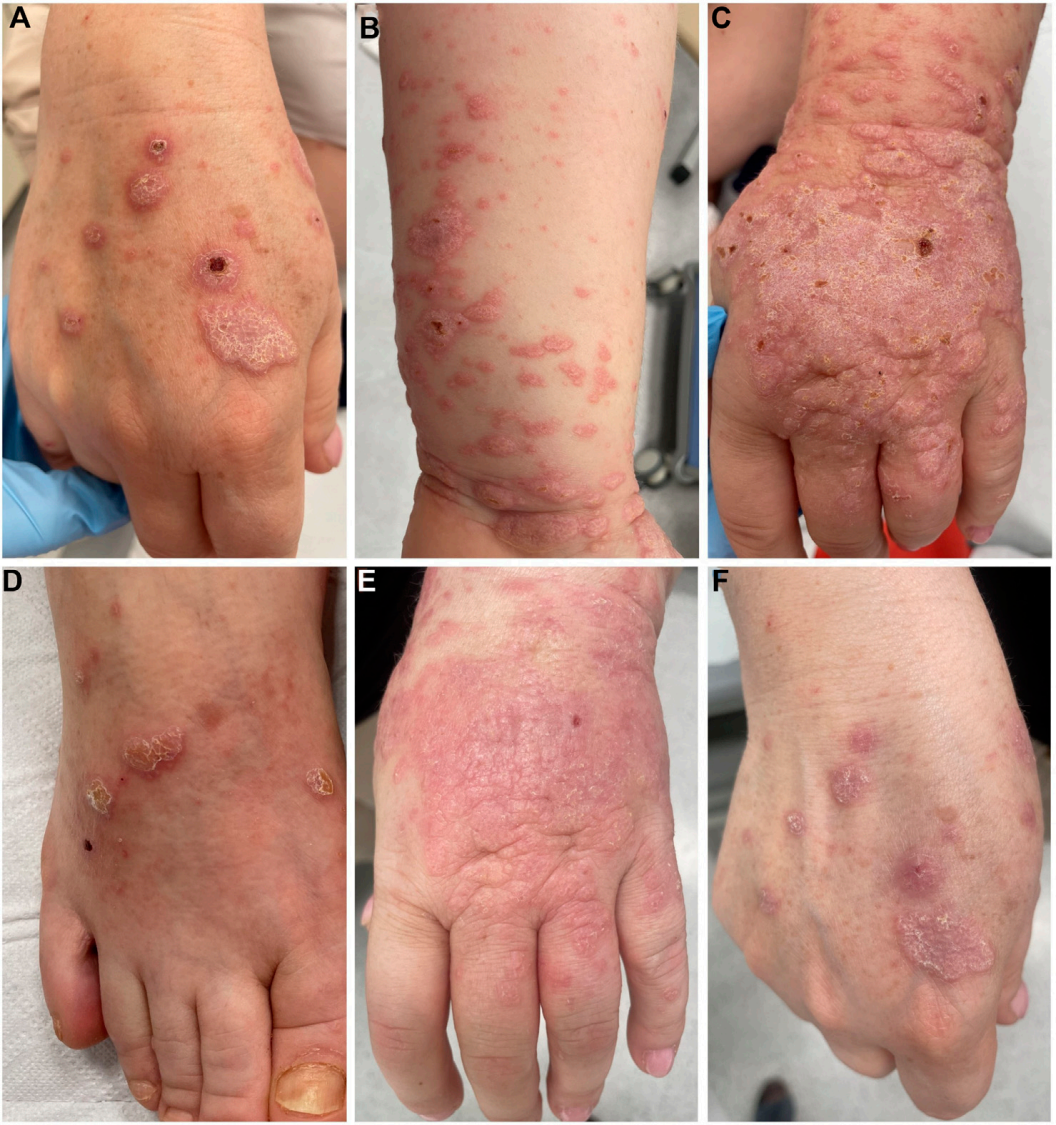
**(A–D)** Patient’s upper and lower limbs upon initial presentation with thick, violaceous, hyperkeratotic plaques with a Wickham’s striae. **(E, F)** Lesions on the patient’s hands after 2 weeks of systemic steroid therapy.

**FIGURE 2:**
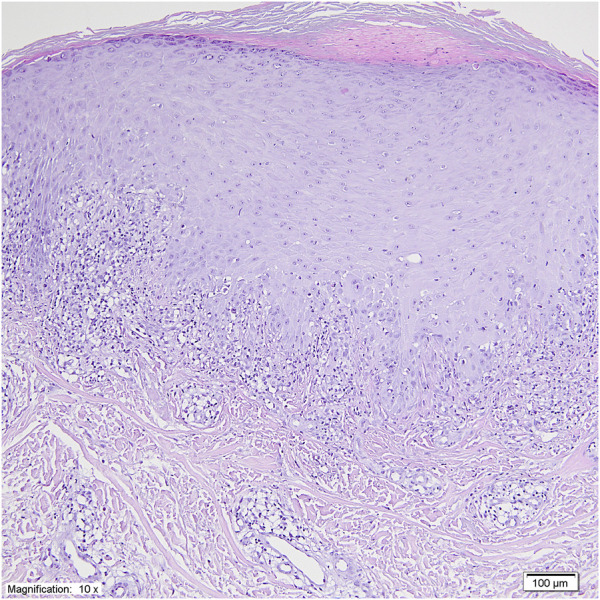
Thickened epidermis with focal parakeratosis, acanthosis and thickening of the granular layer. In the basal layer there is a damage by adjacent, band-like infiltration of small lymphocytes. (H&E staining, magnification × 10).

**FIGURE 3:**
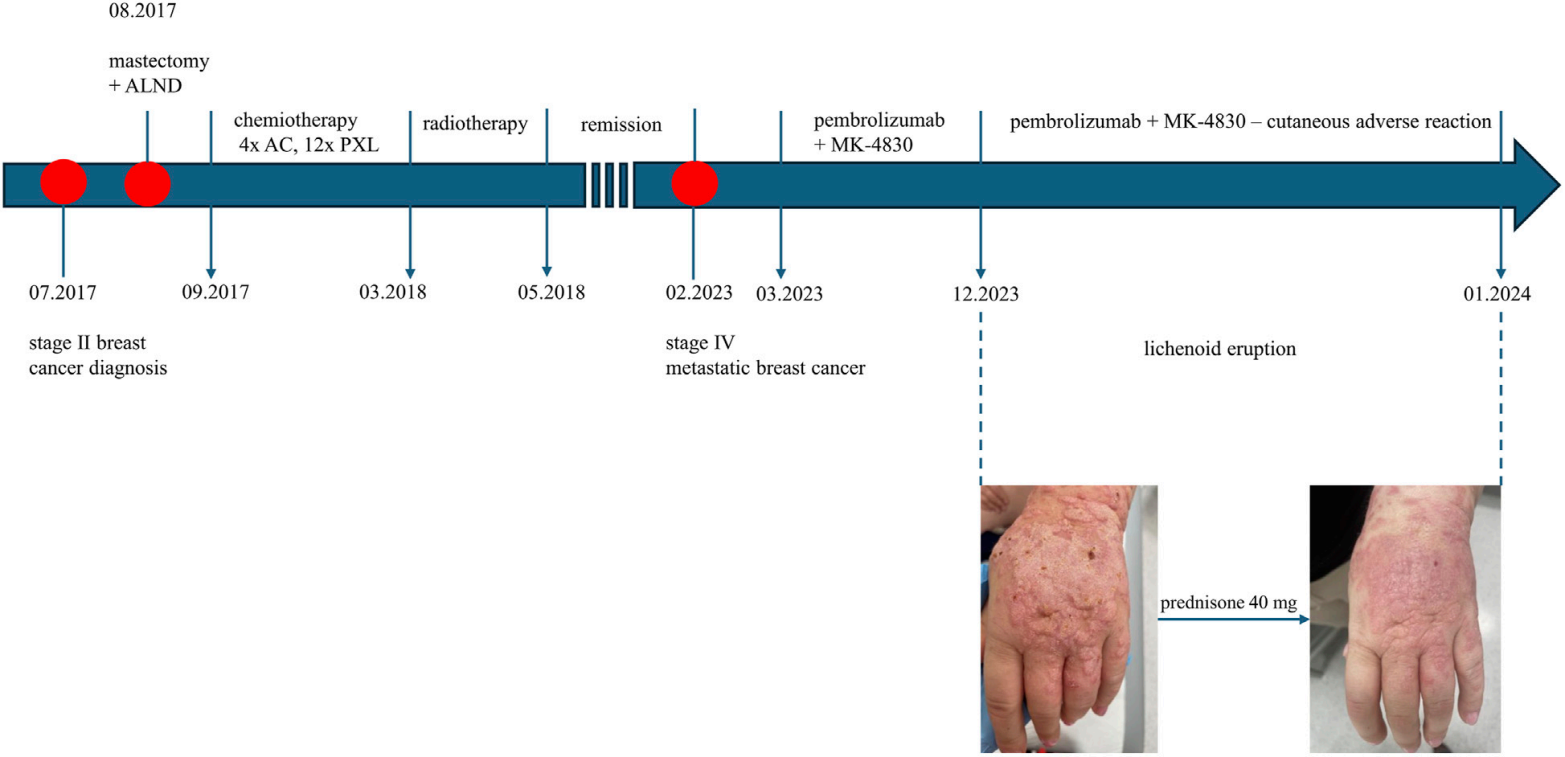
Evolution of clinical events and skin changes. ALND, axillary lymph node dissection; AC, anthracycline-doxorubicine; PXL, paclitaxel.

In July 2017, a 41-year-old Caucasian female was diagnosed with stage IIA pT1cN1T1 breast cancer. Immunohistochemistry: estrogen receptor (ER) (−), progesterone receptor (PR) (−), human epidermal growth factor receptor 2 (HER2) (−), kiel67 antigen (ki-67) positive cells greater than 80%. She underwent left mastectomy and axillary lymph node dissection (ALND) in August 2017. Postoperatively, she received 4 cycles of doxorubicin and 12 cycles of paclitaxel, followed by radiotherapy. Then, regular follow-up was performed. In February 2023, the patient was re-admitted to the hospital due to suspected metastatic spread of breast cancer, as indicated by PET imaging. After relevant examination, metastasis in left lung and neck and mediastinal lymph nodes were considered. The patient received a clinical diagnosis of a stage IV rT0N3M1 breast cancer recurrence. Pembrolizumab and MK-4830 were initiated in March 2023. Tumor staging showed a partial response after 9 months of immunotherapy, as following seven computed tomography (CT) scans indicated a reduction in tumor burden, with a significant decrease in lung and mediastinal metastases, and most importantly, absence of new metastatic lesions. However, a CTCAE (Common Terminology Criteria for Adverse Events) grade 2 drug-induced lichen-planus-like reaction presented at that time with eruption of confluent, flat-topped erythematous papules distributed on upper and lower extremities. The dorsal surfaces of the hands and feet were the most affected areas, exhibiting thick, violaceous, hyperkeratotic plaques with a white, lace-like pattern on the surface (Wickham’s striae) (Figures 1A–D). Histology (biopsy taken from the back of hand) was consistent with lichenoid eruption, showing thickened epidermis with focal parakeratosis, acanthosis and thickening of the granular layer, in the basal layer there was a damage by adjacent, band-like infiltration of small lymphocytes (Figure 2). Examinations of the mucosa, scalp, and nails were unremarkable. All routine blood tests, biochemical analyses, erythrocyte sedimentation rates, coagulation tests, and infection tests yielded negative results. The patient received topical high-potency glucocorticosteroids, which yielded an insufficient response. Subsequently, she received 40 mg of prednisone for 2 weeks, resulting in partial improvement (Figures 1E,F). After that period, the dosage was gradually reduced, providing maintenance therapy with 5 mg of prednisone, which allowed the patient to continue immunotherapy with pembrolizumab and MK-4830 without the necessity of discontinuing the immunotherapy. At the 2-month follow-up, there was no recurrence of skin symptoms. The timeline of this cutaneous adverse reaction and partial skin healing process are summarized in Figure 3.

## Discussion

This study discusses the unique case of a patient with metastatic breast cancer who developed lichenoid lesions after treatment with MK-4830 and pembrolizumab, a phenomenon not previously reported. Among the wide range of irAEs, cutaneous side effects, believed to stem from treatment-related nonspecific hyperactivation of the immune system, are the most commonly reported in patients receiving pembrolizumab (Martin-Liberal et al., 2015). In a study by Sibuad, about one-third of anti-PD-1, including pembrolizumab, treated patients were faced with dermatologic adverse events, which were mostly of immunologic origin, including nonspecific macular papular rash, pruritus, lichenoid dermatitis, psoriasis, Grover’s disease, vitiligo, sarcoidosis, and autoimmune bullous disorders (Sibaud, 2018). Most dermatological toxicities are low grade, primarily grade 1 or 2, according to the CTCAE, which categorizes IRAEs into five grades of escalating severity: asymptomatic/mild (grade 1), moderate (grade 2), severe (grade 3), life-threatening (grade 4), and death (grade 5) (Sibaud, 2018; U.S. DEPARTMENT OF HEALTH AND HUMAN SERVICES, 2023). These toxicities are typically managed with topical emollients, antihistamines, and corticosteroids, including mild to moderate topical corticosteroids and small doses of oral corticosteroids (Darnell et al., 2020). A study by Sanlorenzo et al. confirmed favourable cutaneous safety profile of pembrolizumab, reporting that out of 83 patients who received pembrolizumab, 35 were diagnosed with cutaneous adverse events, with 31 classified as grade 1, two as grade 2, two as grade 3, and none as grade 4^9^.

Data on risk factors in patients receiving ICIs is limited, though it appears that irAEs are more common in women; however, the evidence is conflicting (Alharbi et al., 2022). Also, there may be some genetic predisposition, as Coleman et al. reported that 9% (9/98) of patients who experienced inflammatory eruptions related to ICI therapy had a preexisting dermatosis, most commonly psoriasis, which flared during immunotherapy (Coleman et al., 2019). Furthermore, the mean latency period for inflammatory eruptions varied widely, with granulomatous, lichenoid, psoriasiform, eczematous, and immunobullous reactions generally having longer latency periods compared to maculopapular and Stevens-Johnson syndrome-like eruptions (Coleman et al., 2019; Bhardwaj et al., 2022).

There is also controversy regarding whether the development of cutaneous IrAEs in oncology patients indicates a potential positive prognosis for longer cancer progression-free intervals. A retrospective medical record review by Sanlorenzo et al. (2015) presented survival analyses that showed patients treated with various dosage regimens of pembrolizumab for different cancers had significantly longer progression-free intervals, irrespective of the pembrolizumab treatment regimen. However, we should interpret these findings with caution, as such correlation may apply to patients with grade 1, 2 reactions, but in patients with grade 3–4 cutaneous adverse events, the discontinuation of ICIs for the improvement of irAEs, potentially allow for cancer progression, leading to a poorer prognosis (Bhardwaj et al., 2022).

Lichenoid reactions are inflammatory, T cell mediated reactions to unknown antigen frequently observed with various medications, including ACE inhibitors, beta-blockers, calcium channel blockers, furosemide, thiazide diuretics, and TNF-alpha inhibitors (U.S. DEPARTMENT OF HEALTH AND HUMAN SERVICES, 2023). Moreover, a recent systematic review indicated that lichen planus-like eruptions are the most commonly reported cutaneous adverse event in patients undergoing treatment with ICIs (Alharbi et al., 2022). Clinically, lichenoid eruptions resemble lichen planus, with purplish or pinkish flat-topped papules that are mostly generalised rather than localized, with sparing of mucous membranes and nails. The number of these eruptions can range from tens to hundreds, typically appearing on the trunk, arms, and extremities. The characteristic white lacy fine scales, known as Wickham’s striae, are typically absent. Histologically, these eruptions are characterised by a dense, band-like lymphocytic infiltrate beside the dermal-epidermal junction of the skin, a vacuolar interface, and co-existing spongiosis (Bhardwaj et al., 2022).

Our patient’s punch biopsy with focal parakeratosis suggested a lichenoid drug eruption, contrasting with the typical hyperkeratosis seen in *de novo* lichen planus (Lacouture, 2013). However, the lichenoid eruption observed in our patient differed from the majority of previously reported drug-induced lichen-planus-like cases, primarily due to the presence of Wickham’s striae, which were visible to the naked eye even without dermoscopy. This contrasts with the absence of these white lacy scales typically reported in conventional lichen-planus-like dermatitis (Lacouture, 2013). Although this appearance might suggest the *de novo* development of lichen planus without the influence of immunotherapy with MK-4830 and pembrolizumab, we opted for a lichen-planus-like diagnosis due to the absence of lesions on mucous membranes, nails, and hair, as well as the confluent nature of the lichenoid skin lesions, rather than localized.

The immunologic mechanism of lichenoid dermatitis is supposed to be a T-cell mediated response, with increased self-immunity and pathological reaction to antigens, which results in non-specific T-cell activation. This mechanism could be enhanced by the blockage of PD-1/PD-L1 leading to increased immune response and activation of tumor-specific T-cells (Lee and Seetharamu, 2019). Thirty-five cases of lichenoid dermatitis associated with PD-1/PD-L1 immunotherapy have been reported in publications up to 2022, with sixteen of them being associated with pembrolizumab (Bhardwaj et al., 2022). Interestingly, given the novelty of MK-4830 treatment in oncology, there have been no previously reported cases of lichenoid eruptions, nor the hypothesized pathophysiological mechanism of MK-4830-induced lichenoid dermatitis. In fact, there has only been one singular case describing a cutaneous adverse event in a patient with metastatic squamous cell carcinoma of the head and neck area who developed drug-induced bullous pemphigoid while undergoing immunotherapy with MK-4830 in combination with pembrolizumab (Gresham and Kirchhof, 2023). A first-in-human study conducted by Siu et al., which enrolled 84 oncological patients, with 50 receiving MK-4830 monotherapy and 34 receiving combination therapy with MK-4830 and pembrolizumab, reported that the most common treatment-related adverse events included fatigue, diarrhoea, arthralgia, increased aspartate aminotransferase, decreased appetite, nausea, vomiting, hypothyroidism, and, in the context of dermatology, pruritus (6% of patients in the monotherapy group vs. 4% in the combined therapy group) and maculopapular rash (4% of patients in the monotherapy group vs. 8% in the combined therapy group) (Siu et al., 2022).

Treatment of IRAEs, including lichenoid reactions, primarily follows the CTCAE guidelines, with severity grades based on the percentage of body surface area involved. For grades 1 and 2, conservative management is typically recommended, with the possible use of topical and oral corticosteroids. Grades 3 and 4 often require high doses of oral corticosteroids and may necessitate the use of immunosuppressants. Additionally, for grades 3 and 4, discontinuation of the suspected drug is recommended. Furthermore, it is noteworthy that there is no standardisation for irAEs, and management is based on case reports, case series, and expert consensus (Sibaud, 2018; U.S. DEPARTMENT OF HEALTH AND HUMAN SERVICES, 2023; Bhardwaj et al., 2022; Lee and Seetharamu, 2019). The first-line treatment for lichenoid eruptions subsequent to ICIs involves use of topical and oral steroids, with close monitoring of skin lesions (Sibaud, 2018). Although the majority of published cases of lichenoid eruptions were adequately managed with this approach, some patients with grade 3 reactions required ICI cessation to achieve satisfactory outcomes (Alharbi et al., 2022). In cases of refractory lichenoid eruptions induced by ICIs, narrowband ultraviolet B (NBUVB) phototherapy may be used not only as a steroid-sparing agent but also as a treatment option following the failure of multiple systemic immunosuppressive therapies, including oral corticosteroids (Esfahani et al., 2021).

Our patient initially had an unfavorable response to topical glucocorticosteroids, likely due to reduced penetration in disease-thickened skin and infiltrative nature of lichenoid lesions. Therefore, to address this challenge and enhance the glucocorticosteroids anti-inflammatory effect, a regimen of oral 40 mg prednisone was initiated for a 2-week period, resulting in a significant improvement in the skin lesions. Our patient’s prednisone dosage was determined based on previously reported cases recommending prednisone dosages ranging from 0.5–80 mg/kg for patients with lichenoid eruptions. Given that our patient weighed 60 kg, the 40 mg dosage was appropriate (Bhardwaj et al., 2022). Following this initial treatment, a maintenance therapy with a low dosage of 5 mg prednisone was established, which most importantly, allowed the patient to continue immunotherapy with MK-4830 and pembrolizumab without the need for discontinuation. Furthermore, early recognized cutaneous adverse event and applied treatment resulted in no recurrent skin symptoms in the 2-month follow-up.

Due to the severe prognosis of our patient, she was no longer referred to the dermatology clinic after improvement in skin lesions at the 2-week follow-up. At the 2-month follow-up, she was assessed only by her oncologist, who reported an absence of new skin lesions. Consequently, we lack photographs monitoring her current skin condition, which is the main limitation in our case. Additionally, although temporary use of systemic immunosuppression for the treatment of irAEs appears not to impair the anticancer effects of immune checkpoint inhibitors, there is no unified standard for glucocorticosteroids dosage and treatment duration in ICIs-induced lichenoid eruption, and recommendations relay on limited number of published cases (Postow, 2015).

In conclusion, we describe a first case of MK-4830 and pembrolizumab induced lichenoid reaction in a patient with metastatic breast cancer. We also confirm that lichenoid reactions induced by novel immunotherapeutic agents can be effectively managed by oral corticosteroids. Treatment strategies should be individualized based on each patient’s unique circumstances, considering factors such as the severity of irAEs, future cancer treatment plans, and the tumor’s response to novel immunotherapeutic agents, thereby balancing the risks and benefits of both irAEs management and oncology treatment options. As long as a patient does not present with grade 4 reaction, there should be at least attempts to treat them without necessity of immunotherapy discontinuation.

## Patient perspective

The patient was pleased that treatment with oral prednisone allowed her to successfully continue treatment with MK-4830 and pembrolizumab, which she hoped would maintain cancer remission. She was grateful for the improvement in her skin lesions, which resulted in a better quality of life.

## Data Availability

The original contributions presented in the study are included in the article/supplementary material, further inquiries can be directed to the corresponding author.
